# Melatonin Alleviates Silica Nanoparticle-Induced Lung Inflammation via Thioredoxin-Interacting Protein Downregulation

**DOI:** 10.3390/antiox10111765

**Published:** 2021-11-04

**Authors:** Je-Oh Lim, Se-Jin Lee, Woong-Il Kim, So-Won Pak, Jong-Choon Kim, Joong-Sun Kim, Young-Kwon Cho, In-Chul Lee, In-Sik Shin

**Affiliations:** 1 College of Veterinary Medicine and BK21 FOUR Program, Chonnam National University, Gwangju 61186, Korea; 166634@jnu.ac.kr (J.-O.L.); 196433@jnu.ac.kr (S.-J.L.); 208521@jnu.ac.kr (W.-I.K.); 208514@jnu.ac.kr (S.-W.P.); toxkim@jnu.ac.kr (J.-C.K.); centraline@jnu.ac.kr (J.-S.K.); 2 College of Health Sciences, Cheongju University, Chungbuk 28503, Korea; petmen@hanmail.net; 3 Functional Biomaterial Research Center, Korea Research Institute of Bioscience and Biotechnology, Jeonbuk 56212, Korea

**Keywords:** melatonin, silica dioxide nanoparticle, lung inflammation, human airway epithelial cell line, thioredoxin-interacting protein

## Abstract

Silica dioxide nanoparticles (SiONPs) have been increasingly used in various industries; however, this has raised concerns regarding their potential toxicity. SiONPs are also a major component in the Asian sand dust that causes pulmonary diseases among the general public. Melatonin exerts some inhibitory effects against lung inflammation. In this study, we explored the therapeutic properties of melatonin against lung inflammation using an SiONPs-induced lung inflammation murine model and SiONPs-stimulated H292 cells, human airway epithelial cell line, by focusing on the involvement of thioredoxin-interacting protein (TXNIP) in the modulation of the MAPKs/AP-1 axis. We induced an inflammatory response by exposing mouse lungs and the H292 cells to SiONPs and confirmed the anti-inflammatory effect of melatonin. Melatonin inhibited the expression of various inflammatory mediators, including *TNF-α*, *IL-6*, and *IL-1β*, in SiONPs-exposed mice and SiONPs-stimulated H292 cells; this inhibition contributed to a decline in inflammatory cell accumulation in the lung tissues. Furthermore, melatonin treatment decreased the expression of MAPKs and AP-1 by downregulating TXNIP, eventually decreasing the production of SiONPs-induced inflammatory mediators. Overall, these data suggest that melatonin reduces SiONPs-induced lung inflammation by downregulating the TXNIP/MAPKs/AP-1 signalling pathway, thereby supporting the use of melatonin as an effective approach to control SiONPs-induced lung inflammation.

## 1. Introduction

Silica dioxide nanoparticles (SiONPs) have been extensively used in biotechnology owing to the simple production process and ability to modify their shape and size [1]. The increasing use of SiONPs has raised concerns regarding their potential toxicity in humans. This is particularly true in an industrial setting where SiONPs can be inhaled and can be linked to serious health problems, including lung cancer, silicosis, emphysema, and chronic obstructive pulmonary disease (COPD) [2,3]. Inhaled SiONPs induce significant pulmonary inflammation, which can result in the activation of the MAPKs [4,5,6,7]. These responses further induce the activation of AP-1, resulting in accelerated inflammatory processes [8]. Furthermore, SiONPs are a major component in the Asian sand dust that affects northeast Asian countries, including China, Japan and Korea, especially in the spring, and are associated with the occurrence of various pulmonary diseases across this region [9]. Thus, controlling the respiratory diseases caused by SiONPs is critical to maintaining human health.

Thioredoxin-interacting protein (TXNIP), an endogenous inhibitor of thioredoxin, is closely involved in the regulation of various inflammatory responses [10]. TXNIP contributes to the inflammation of the respiratory tract by interacting with the upregulated MAPKs pathway [11,12,13,14]. Recent studies have reported that exposure to SiONPs elevates the TXNIP expression, activating MAPKs and increasing pulmonary inflammation [15,16]. Therefore, inhibiting TXNIP/MAPKs signalling may be a potential strategy for treating SiONPs-induced pulmonary inflammation.

Melatonin is primarily released by the pineal gland and is known to exert a wide range of biological properties, such as anti-inflammatory and antioxidant properties [17,18,19]. Consequently, melatonin has been shown to exhibit some therapeutic effects on various respiratory diseases [20,21]. Exogenous melatonin alleviates inflammatory response by inhibiting MAPK signalling in the respiratory tract of asthmatic mice and attenuates smoke-induced pulmonary inflammation by inhibiting Erk-Sp1 expression. Furthermore, the anti-inflammatory effects of melatonin are associated with inhibition of TXNIP, as established in a cadmium-induced liver injury model [22]. However, the underlying mechanism of anti-inflammatory effects of melatonin against SiONPs-induced pulmonary inflammation via TXNIP regulation is not comprehensively understood.

The goals of this study were to explore the anti-inflammatory effect of melatonin on SiONPs-induced pulmonary inflammation and to elucidate the underlying mechanism of this hormone by focusing on its effects on TXNIP/MAPKs signalling.

## 2. Materials and Methods

### 2.1. SiONPs

The particle size of SiONPs (Sigma-Aldrich, St. Louis, MO, USA) was <5–15 nm. It was dissolved with phosphate-buffered saline (PBS) and sonicated (VCX-130; Sonics and Materials) for 3 min before treatment.

### 2.2. Cell Culture

The human airway epithelial cells (NCI-H292, ATCC) were cultured with RPMI 1640 medium (WELGENE, Gyeongbuk, Korea) added to foetal bovine serum (10%), streptomycin (100 U/mL) and penicillin (100 μg/mL) and were grown in a humidified incubator maintained at 37 °C with 5% CO_2_. The cells were serum-starved for 1 h before use.

### 2.3. Real-Time PCR

Cells were seeded into 60 mm dishes (8 × 10^5^ cells/dish) and grown for 24 h and treated with 50, 100, 200, and 400 μM melatonin for 12 h. The cells were treated with SiONPs (12.5 μg/mL) for 6 h. Total RNA was separated using the TRIzol reagent (Invitrogen) and reverse transcribed using a cDNA kit (Qiagen, Hilden, Germany). Information of specific primers is described in Table 1. Real-time PCR experiment conditions were as follows: 15 min at 95 °C, 20 s at 95 °C/40 s at 55 °C for 40 cycles, and 10 s at 95 °C/5 s at 65 °C/60 s at 95 °C for the melting curve. The qRT-PCR reaction system was 20 µL: SYBR Premix I (Biofact, Daejeon, Korea), 10 µL; PCR Forward Primer (10 µM), 1 µL; PCR Reverse Primer (10 µM), 1 µL; cDNA template, 2 µL; and distilled water, 6 µL. The mRNA expressions of *TNF-α, IL-6*, and *IL-1β* were calculated by the 2^−^^∆∆^^CT^ method with the internal reference as *GAPDH*.

### 2.4. Ethics Statement

The Institutional Animal Care and Use Committee at the Chonnam National University approved the protocols used in this animal study (CNU IACUC-YB-2019-63).

### 2.5. Preparation of Animals and Experimental Design

Female BALB/c mice (6 weeks old, Samtako Co., Gyeonggido, Korea) were placed in quarantine for one week and allowed to acclimate. The animals were fed a laboratory diet and water ad libitum. The mice were randomized into five groups (*n* = 6 per group); the (1) normal control (NC), (2) SiONPs (SiONPs only), (3) dexamethasone (DEX, SiONPs + 2 mg/kg DEX), (4) Mel 20 (SiONPs + 20 mg/kg melatonin), and (5) Mel 40 (SiONPs + 40 mg/kg melatonin) groups. DEX and melatonin were administered using oral gavage over two weeks. SiONPs (20 mg/kg in 50 µL of PBS) were intranasally instilled three times (days 1, 7 and 13) under anaesthesia. The NC group received PBS (50 µL) via the same route.

### 2.6. Inflammatory Cell Count in Bronchoalveolar Lavage Fluid (BALF)

Mice were sacrificed 48 h post final SiONPs exposure and were subjected to tracheostomy. The BALF was obtained by infusing the lungs with cold PBS (0.7 mL) two times, which was subsequently collected using a tracheal cannula. Differential cell counts in the BALF were determined using a Diff-Quik^®^ reagent (IMEB Inc., San Marcos, CA, USA) as described by the manufacturer.

### 2.7. Enzyme-Linked Immunosorbent Assay (ELISA)

Expression of cytokines, including interleukin *(IL)-1β*, *IL-6*, and tumour necrosis factor-α (*TNF-α*), in the BALF were quantified using ELISA kits (BD Biosciences, San Jose, CA, USA).

### 2.8. Histological Examination and Immunohistochemistry (IHC)

To estimate the degree of inflammation, the lung tissues fixed with paraformaldehyde (4% *v*/*v*) were stained with H&E (Sigma-Aldrich, St. Louis, MO, USA) after going through a series of procedures. The degree of inflammation was quantified using an image analyser (IMT i-Solution, Vancouver, BC, Canada). The IHC examinations were performed as previously described [20]. The anti-AP-1 (ab21981; 1:200; Abcam) and anti-TXNIP (NBP1-54578; 1:200; Novus Biologicals, Littleton, CO, USA) were used as primary antibodies.

### 2.9. Western Blot Analysis

Western blot analysis was conducted as previously described [21]. Phospho-ERK1/2 (#9101), total ERK (#9102), phosphor-JNK (#9251), total JNK (#9252), phosphor-p38 (#4631), total p38 (#9212) and β-actin (#4967) were obtained from Cell Signalling. TXNIP (NBP1-54578) and AP-1 (ab21981) were purchased from Novus Biologicals and Abcam, respectively. Protein expression was measured by a ChemiDoc system (Bio-Rad, Hercules, CA, USA).

### 2.10. Double-Immunofluorescence and Confocal Microscope

Double-immunofluorescence was performed as previously described [23], using anti-AP-1 (ab21981; 1:200; Abcam) and anti-TXNIP (NBP1-54578; 1:200; Novus Biologicals) antibodies, and imaging was completed using a Leica TCS SP5 AOBS laser scanning confocal microscope (Leica Microsystems, Hesse, Germany) under a Leica 63× (N.A. 1.4) oil objective.

### 2.11. siRNA Transfection

Small interfering RNAs (siRNA) against the scrambled siRNA (4390843) control and TXNIP (4392420) were obtained from Ambion. Each siRNA (20 nM) was transfected into cells using Lipofectamine^TM^ RNAiMAX (Invitrogen, Carlsbad, CA, USA) via the forward transfection method, as prescribed by the manufacturer. After suppression of endogenous TXNIP expression, cells were treated with 12.5 µg/mL SiONPs or PBS and harvested after 6 h. To investigate the protein expression, Western blot was performed as mentioned above.

### 2.12. Statistical Analysis

Data are presented as the means ± standard deviation. All statistical analyses were performed using GraphPad Prism 7 (San Diego, CA, USA). One-way analysis of variance (ANOVA) was performed, followed by the Bonferroni multiple comparison test. *p* < 0.05 was considered significant.

## 3. Results

### 3.1. Melatoinin Reduces the Number of Inflammatory Cells of the BALF from SiONPs-Exposed Mice

**Figure 1:**
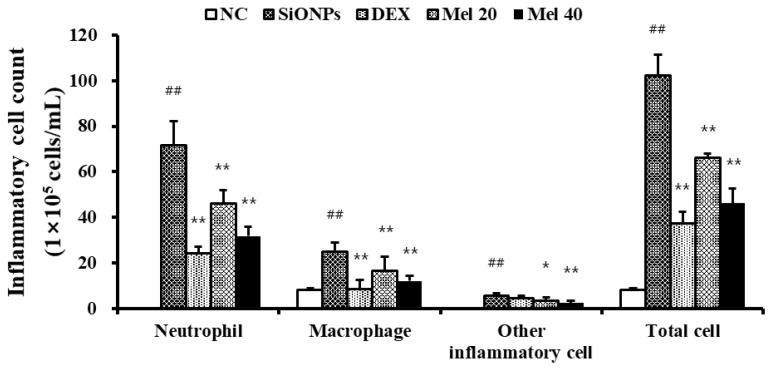
Melatonin decreased inflammatory cell count in SiONPs-exposed mice. Particularly, melatonin reduced the elevated neutrophils and macrophages of BALF in SiONPs-exposed mice. ^##^ *p* < 0.01, vs. the NC; * *p* < 0.05, ** *p* < 0.01, vs. the SiONPs.

Exposure to SiONPs showed markedly higher inflammatory cell counts of BALF when compared with those in the NC group (Figure 1), with neutrophils and macrophages exhibiting significant elevation. DEX-treated animals exhibited lesser inflammatory cells than those in the SiONPs-exposed specimens. Moreover, there was a significant decrease in the number of inflammatory cells in the melatonin-treated animals compared with those in the SiONPs-exposed group, as observed in 40 mg/kg of melatonin (Figure 1).

### 3.2. Melatonin Decreases Inflammatory Cytokines of the BALF from SiONPs-Exposed Mice

**Figure 2:**
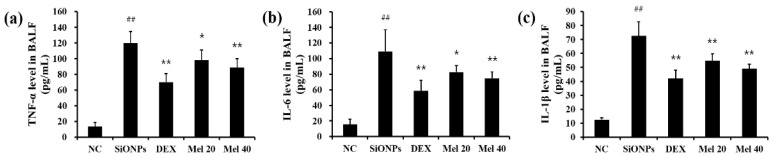
Melatonin reduced the levels of cytokine in SiONPs-exposed mice. (**a**) *TNF-α*, (**b**) *IL-6*, and (**c**) *IL-1β* levels in BALF. Cytokine levels were measured using an ELISA. ^##^ *p* < 0.01, vs. the NC; * *p* < 0.05, ** *p* < 0.01, vs. the SiONPs.

Exposure to SiONPs significantly elevated the *TNF-α* (119.64 ± 14.9 pg/mL, *p* < 0.01) and *IL-6* (109.18 ± 27.5 pg/mL, *p* < 0.01) levels in BALF in comparison with those in the NC group (*TNF-α*: 13.50 ± 5.21 pg/mL, *IL-6*: 15.64 ± 6.68 pg/mL) (Figure 2a,b, respectively). However, both the DEX- and melatonin-treated groups exhibited marked declining in the levels of *TNF-α* (DEX: 70.07 ± 11.0 pg/mL, *p* < 0.01; Mel 20: 98.01 ± 13.2 pg/mL, *p* < 0.05; Mel 40: 88.57 ± 11.2 pg/mL, *p* < 0.01) and *IL-6* (DEX: 58.49 ± 13.8 pg/mL, *p* < 0.01; Mel 20: 82.69 ± 8.07 pg/mL, *p* < 0.05; Mel 40: 74.62 ± 8.13 pg/mL, *p* < 0.01) in comparison with those in the SiONPs group (Figure 2a,b, respectively). Additionally, the increased *IL-1β* (72.56 ± 10.1 pg/mL) levels in the SiONPs group significantly decreased following treatment with melatonin (Mel 20: 54.81 ± 4.93 pg/mL, *p* < 0.01; Mel 40: 49.10 ± 3.21 pg/mL, *p* < 0.01) (Figure 2c).

### 3.3. Melatonin Ameliorates Pathological Changes in the Respiratory Tract of SiONPs-Exposed Mice

**Figure 3:**
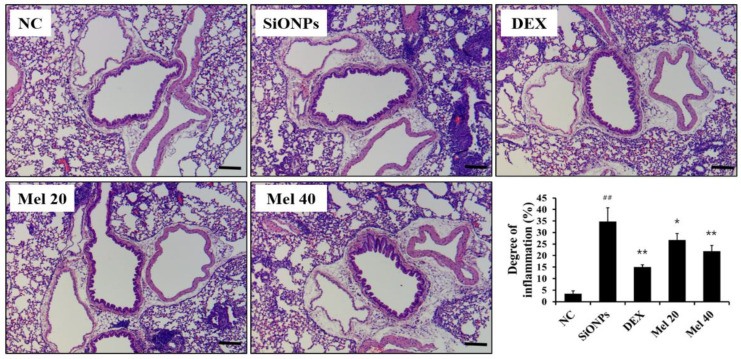
Melatonin ameliorated inflammatory responses in the respiratory tract of SiONPs-exposed mice. Lung tissue was stained with H&E stain to assess airway inflammation (magnification: x200). Degree of inflammation was quantified using image analyzer (IMT i-Solution). Scale bars = 50 μm. ^##^ *p* < 0.01, vs. the NC; * *p* < 0.05, ** *p* < 0.01, vs. the SiONPs.

Exposure to SiONPs noticeably increased inflammatory cell accumulation (34.70 ± 6.03%, *p* < 0.01) in the respiratory tract as compared with that in the NC group (3.37 ± 1.22%) (Figure 3). However, the accumulation in these tissues markedly declined in the DEX-treated mice (15.02 ± 1.04%, *p* < 0.01) in comparison with that in the SiONPs group. Melatonin-treated animals also exhibited a notable decrease in inflammatory cell accumulation (Mel 20: 26.73 ± 2.75%, *p* < 0.05) in comparison with that in the SiONPs group, which was also evident in the 40 mg/kg melatonin specimens (Mel 40: 21.80 ± 2.59%, *p* < 0.01).

### 3.4. Melatonin Inhibits TXNIP/MAPK/AP-1 Pathway in the Lungs of SiONPs-Exposed Mice

**Figure 4:**
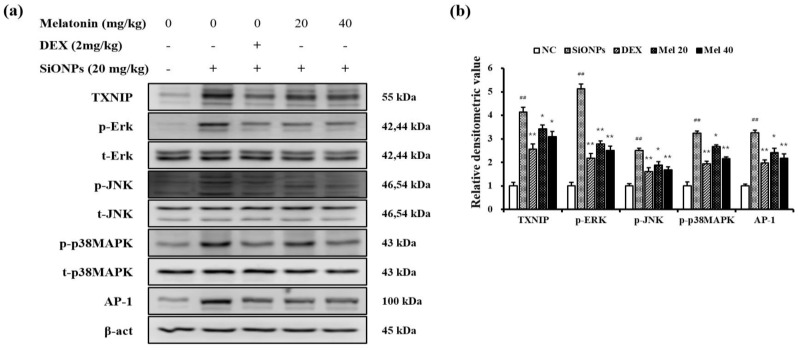
Melatonin suppressed the activation of TXNIP/MAPK/AP-1 pathway in SiONPs-exposed mice. (**a**) Representative figure for Western blot, (**b**) densitometric value. Densitometric expression value of each protein was quantified using Chemi-Doc (Bio-rad). ^##^ *p* < 0.01, vs. the NC; * *p* < 0.05, ** *p* < 0.01, vs. the SiONPs.

Exposure to SiONPs resulted in a clear increase in TXNIP expression (Figure 4a,b). However, DEX- and melatonin-treated mice exhibited a notable decline in TXNIP expression compared with that on SiONPs exposure. Additionally, SiONPs exposure markedly elevated phosphorylation of ERK, JNK, and p38, which was accompanied with an increase in the AP-1 expression. However, these significantly decreased following melatonin treatment. These observations were also consistent with those of the TXNIP expression assays.

### 3.5. Melatonin Inhibits the Expression of TXNIP and AP-1 in Respiratory Tract of SiONPs-Exposed Mice

**Figure 5:**
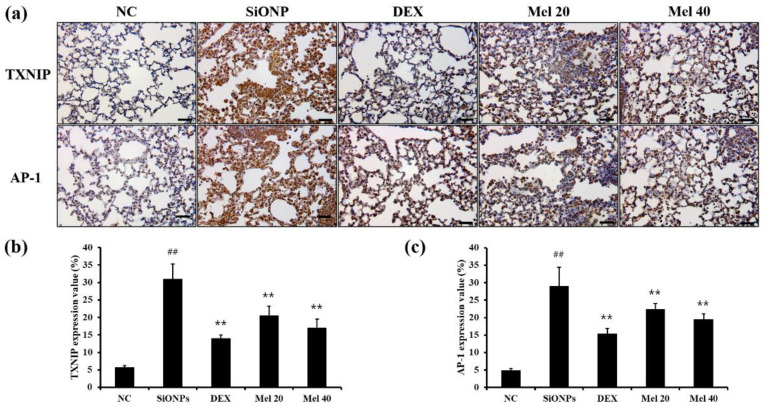
Melatonin suppressed the expression of TXNIP and AP-1 in the respiratory tract of SiONPs-exposed mice. (**a**–**c**) IHC was performed to measure the expression of TXNIP (1:200) and AP-1 (1:200). Quantitative analysis of each protein expression was conducted using image analyser (IMT i-Solution) (×200). Scale bars = 50 μm. ^##^ *p* < 0.01, vs. the NC; ** *p* < 0.01, vs. the SiONPs.

SiONPs exposure resulted in elevated expression of TXNIP (31.03 ± 4.34%, *p* < 0.01) and AP-1 (29.03 ± 5.31%, *p* < 0.01) in the respiratory tract as compared with that in the NC group (TXNIP: 5.73 ± 0.51%, AP-1: 4.93 ± 0.49%) (Figure 5a–c). However, DEX-treated mice exhibited decreased expression of TXNIP (14.02 ± 0.97%, *p* < 0.01) and AP-1 (15.36 ± 1.52%, *p* < 0.01) compared with that observed in the SiONPs group; this trend was also observed for the 40 mg/kg melatonin-treated animals, which exhibited the most significant decrease in expression of TXNIP (Mel 20: 20.57 ± 2.68%, *p* < 0.01, Mel 40: 17.13 ± 2.38%, *p* < 0.01) and AP-1 (Mel 20: 22.40 ± 1.65%, *p* < 0.01; Mel 40: 19.47 ± 1.66%, *p* < 0.01).

### 3.6. Melatonin Attenuates mRNA Expression of Inflammatory Cytokines in SiONPs-Stimulated Cells

**Figure 6:**
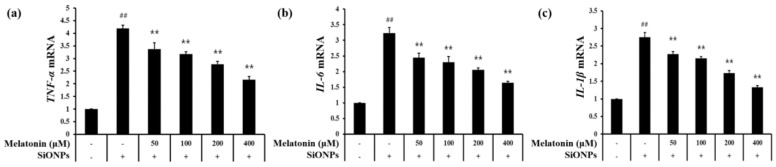
Melatonin suppressed the mRNA expression of inflammatory cytokines in SiONPs (12.5 μg/mL)-stimulated cells as assessed using real-time PCR. (**a**) *TNF-α* (**b**) *IL-6*, (**c**) *IL-1β* (*n* = 3). ^##^ *p* < 0.01, vs. non-exposed cells; ** *p* < 0.01, vs. SiONPs-stimulated cells.

The *TNF-α* and *IL-6* mRNA expression were markedly higher in the SiONPs-stimulated cells than in non-stimulated cells (Figure 6a,b, respectively). However, melatonin treatment reduced this increase in a concentration-dependent manner. Additionally, the elevated *IL-1β* mRNA expression in the SiONPs markedly decreased following treatment with melatonin (Figure 6c).

### 3.7. Melatonin-Mediated TXNIP Inhibition Reduces the MAPKs Phosphorylation and AP-1 Expression in SiONPs-Stimulated Cells

**Figure 7:**
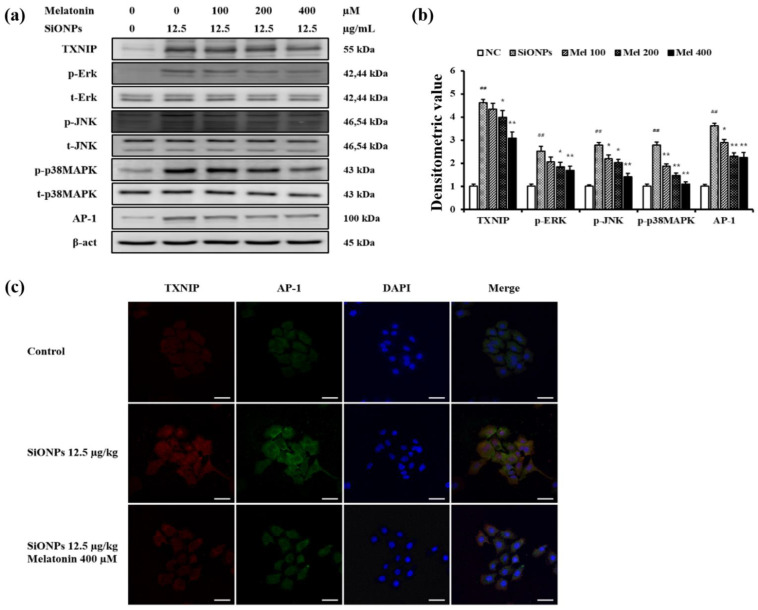
Melatonin inhibited the activation of TXNIP/MAPK/AP-1 pathway in SiONPs-stimulated cells. (**a**) Representative figure for Western blot, (**b**) densitometric value, (**c**) immunofluorescence for TXNIP and AP-1. Densitometric expression value of each protein was quantified using Chemi-Doc (Bio-rad). Scale bars = 25 μm (*n* = 3). ^##^ *p* < 0.01, vs. non-exposed cells; * *p* < 0.05, ** *p* < 0.01, vs. SiONPs-stimulated cells.

SiONPs treatment markedly elevated TXNIP expression (Figure 7a–c). However, melatonin treatment suppressed TXNIP expression in SiONPs-stimulated cells when compared with that in non-stimulated cells, which was accompanied with increases in JNK, ERK, and p38 MAPK phosphorylation. However, this reduced following the addition of melatonin, consistent with the TXNIP expression trend (Figure 7a,b). Furthermore, SiONPs treatment noticeably elevated AP-1 expression, which was declined following the melatonin treatment (Figure 7a–c).

**Figure 8:**
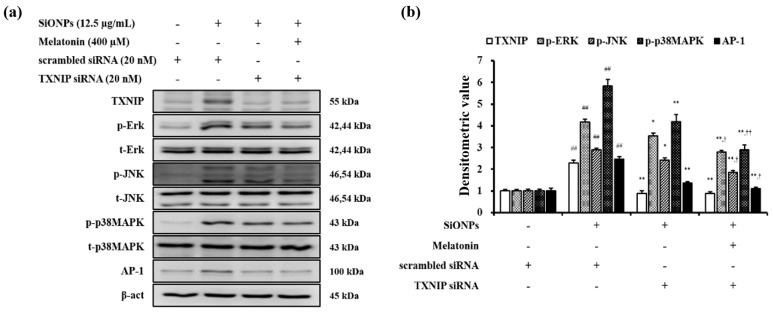
Melatonin suppressed inflammatory signalling in SiONPs-stimulated cells via TXNIP downregulation. (**a**) Representative figure for Western blot, (**b**) densitometric value. Densitometric expression value of each protein was quantified using Chemi-Doc (Bio-rad) (*n* = 3). ^##^ *p* < 0.01 vs. scrambled siRNA-transfected cells; * *p* < 0.05, ** *p* < 0.01 vs. SiONPs-stimulated cells; ^†^ *p* < 0.05, ^††^ *p* < 0.01 vs. TXNIP siRNA-transfected cells stimulated by SiONPs.

TXNIP or MAPK expression was not affected by the control siRNA, whereas their expression was downregulated by the TXNIP siRNA (Figure 8a,b). Furthermore, melatonin and TXNIP siRNA together demonstrated an even more pronounced reduction in TXNIP expression and the MAPKs phosphorylation in SiONPs-stimulated cells than TXNIP siRNA treatment alone.

## 4. Discussion

SiONPs have various industrial applications owing to their many advantages, but their increasing use has proven detrimental to the health of workers dealing with them [2,3]. Furthermore, SiONPs are a major constituent of Asian sand dust; thus, they are the underlying cause of increasing susceptibility to bacteria or viral infections in populations exposed to Asian sand dust [9]. In recent studies, SiONPs were shown to induce changes in the immune function of exposed cell; in particular, exposure to these particles has been linked to excessive inflammation in the respiratory tract [4,6,7,24]. Conversely, melatonin has exhibited protective effects against asthma and COPD owing to its pharmacological properties [17,20,21,25]. Based on literature, we investigated the activity of melatonin on SiONPs-induced pulmonary inflammation using both experimental animal models and cell lines. In this study, melatonin effectively attenuated SiONPs-induced pulmonary inflammation and this anti-inflammatory effect was linked to the downregulation of the TXNIP/MAPK/AP-1 pathway.

Excessive inflammatory responses result in the development and exacerbation of lung injuries due to the production of inflammatory cytokines [26,27]. SiONPs increase inflammatory cell infiltration and cytokine expression levels by activating inflammatory signalling, resulting in lung injury [7,16]. Therefore, a reduction in the inflammatory responses induced by SiONPs may offer a potential therapeutic strategy for preventing and treating SiONPs-related lung damage. The therapeutic effects of melatonin against inflammatory responses have been reported by several experimental studies, which suggest that melatonin is effective in preventing lung damage associated with enhanced inflammatory responses [20,21,28,29,30]. Several studies have shown that melatonin reduces inflammatory mediators produced in response to various stimuli including radiation, carbon tetrachloride, and allergens, thereby preventing lung injury [28,29,31]. Moreover, melatonin treatment alleviates the pulmonary inflammation associated with cigarette smoke-induced COPD by suppressing MAPK phosphorylation [21]. In this study, SiONPs induced the inhibition of several inflammatory mediators as previously reported [15,16]. However, treatment with melatonin reduced the inflammatory index, including those of inflammatory cells and cytokines, in both the SiONPs-exposed mice and SiONPs-stimulated cells. These changes were accompanied by histological evidence of a decrease in inflammatory cell infiltration in the respiratory tract. Overall, these results indicate that melatonin can act as a therapeutic agent and prevent lung damage caused by exposure to SiONPs.

TXNIP has been implicated in the development of many biological reactions, including inflammatory responses [10]. TXNIP overexpression in response to various stimuli induces the phosphorylation of various MAPKs including p38 and JNK, whereas silencing of TXNIP suppresses the activation of MAPK signalling [32,33]. This increase in MAPK phosphorylation increases AP-1 activation, leading to an increase in inflammatory mediators and consequently making TXNIP an important regulator of the inflammatory responses [34,35]. Exposure to SiONPs induces airway inflammation in experimental animals, and these increases were associated with an elevation in TXNIP expression levels [15]. However, silibinin, an antioxidant and anti-inflammatory agent, suppressed the pulmonary inflammation caused by SiONP exposure by inhibiting TXNIP expression [16]. Thus, TXNIP inhibitors may effectively suppress the lung inflammation associated with SiONP exposure. In this study, exposure to SiONPs caused pulmonary inflammation and activated the TXNIP/MAPK/AP-1 pathway in mice. However, in both the experimental animal models and H292 cell lines, melatonin reduced the activation of TXNIP/MAPK/AP1 signalling associated with SiONP exposure, resulting in a decline in the production of inflammatory mediators and the eventual reduction of lung inflammation, thus indicating that melatonin acts as a TXNIP inhibitor and can effectively inhibit the lung inflammation associated with SiONPs.

## 5. Conclusions

Our data show that melatonin suppresses the SiONP-induced inflammatory responses in both the mouse lung tissues and H292 cells. These effects were closely associated with the downregulation of the TXNIP/MAPK/AP-1 pathway, allowing us to establish that melatonin effectively inhibits SiONP-induced lung inflammation by downregulating TXNIP expression.

## Figures and Tables

**Table 1:** Primer sequences for real-time PCR.

Target Genes	Forward Primer (5′→3′)	Reverse Primer (5′→3′)
*TNF-α*	CAA AGT AGA CCT GCC CAG AC	GAC CTC TCT CTA ATC AGC CC
*IL-6*	ATG CAA TAA CCA CCC CTG AC	ATC TGA GGT GCC CAT GCT AC
*IL-1β*	AGC CAG GAC AGT CAG CTC TC	ACT TCT TGC CCC CTT TGA AT
*GAPDH*	CAA AAG GGT CAT CAT CTC TG	CCT GCT TCA CCA CCT TCT TG

## Data Availability

The data presented in this study are available in this manuscript.
